# Effect of *Plantago ovata* Forsk seed mucilage on survivability of *Lactobacillus acidophilus*, physicochemical and sensory attributes of produced low‐fat set yoghurt

**DOI:** 10.1002/fsn3.2074

**Published:** 2020-12-23

**Authors:** Seyedeh Zahra Mehrinejad Choobari, Abbas Ali Sari, Amir Daraei Garmakhany

**Affiliations:** ^1^ Department of food hygiene and quality control Faculty of veterinary science Bu‐Ali Sina University Hamedan Iran; ^2^ Department of Food Science and Technology Tuyserkan Faculty of Engineering & Natural Resources Bu‐Ali Sina University Hamedan Iran

**Keywords:** *Lactobacillus acidophilus*, Low‐fat yoghurt, mucilage, Physicochemical and sensory attributes, *Plantago ovata* Forsk

## Abstract

Nowadays, consumers’ attention to the functional foods has increased significantly. In this study, the effect of different concentration (0.5, 1, and 2%) of *P.ovata* Forsk seed mucilage (PFM) on survivability of *L.acidophilus*, physicochemical, and sensory attributes of produced low‐fat yoghurt were investigated in 0, 7, 14, and 21 days of storage period. Results showed that at the beginning of the storage period, the number of *L.acidophilus* in yoghurt samples containing PFM was significantly higher than control sample. The highest number of *L.Acidophilus* was observed in yoghurt sample contain 2% PFM (6.68 log CFU/g) on the first day of storage period. The lowest decrease of *L.Acidophilus* (0.2 log CFU/g) was observed in the sample contain 2% PFM. Treatments containing PFM had lower pH and higher acidity than the control sample. Addition of PFM to the yoghurt samples increased water holding capacity (WHC) during storage period significantly while syneresis decreased. The highest WHC (89%) and the lowest syneresis (6%) were observed in yoghurt sample containing 2% PFM. Sensory evaluation results showed that the treatments containing PFM were not significantly different in taste, but the probiotic yogurt containing 1% PFM had the highest acceptability in terms of total appearance and texture. Evaluation of L, a, and b values indicated that yoghurt sample containing 2% PFM was significantly lower in L and b values and higher in a value than the control sample. Therefore, using *P.ovata* Forsk seed mucilage in yoghurt sample formulation improved the physicochemical attributes and probiotic survivability of produced yoghurt sample.

## Graphical abstract

In this study, the effect of different concentrations of P.ovata Forsk seed mucilage was investigated on survivability of L.acidophilus and physicochemical and sensory properties of low‐fat yoghurt.

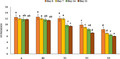

## 1 INTRODUCTION

Nowadays, new eating habits and paying more attention to prevent diseases by healthy diet have led to the creation of functional foods due to their effect on preventing gastrointestinal and cardiovascular disease or different cancers. Many improvements in the production of this type of foods have been achieved by producing probiotic products and the addition of some soluble fibers that called prebiotics (de Souza Oliveira et al., 2011). Probiotics are live and beneficial microorganisms which transit the gastrointestinal tract and have benefits for the health of consumers (Tannock et al., 2000). Taking probiotic products have good effects like improving immune system performance, controlling serum cholesterol level, preventing intestinal infection, improving lactose consumption in patients with lactose intolerance disease and the anticarcinogenic role (Gilliland, 1990; Parvez et al., 2006). A probiotic product must contain at least 10^6^–10^8^ CFU/g live microorganism at the time of consumption (Michael et al., 2015; Minelli & Benini, 2008). The most common probiotic food carriers to human bodies are yoghurt and fermented milk, because these products have high nutritional value and are highly accepted and popular among consumers (Antunes et al., 2007; Shah, 2000). Different quality attributes of yoghurt, such as sensorial, texture, rheological, and microstructural properties, are dependent to the several factors, including fermentation procedure, type of milk and starter culture, packaging process, and storage condition (Murphy et al., 2016). Most studies in the production of probiotic foods have been done by using the genera *Lactobacillus* sp. and *Bifidobacterium* sp. in recent years. To receive their advantages, the probiotics must not only keep their viability in different adverse condition such as production process, storage condition, the gastric environment, hydrolytic enzymes, and bile salts from the gastrointestinal tract, but also have no negative effects on the sensory and physicochemical properties of the product (Ding & Shah, 2007; Liu et al., 2007).

Prebiotics are compounds that are commonly used to increase the viability of bacteria and keep their survival until consumption (Gibson & Roberfroid, 1995). The combination of probiotics and prebiotics produces synbiotic which are useful and beneficial products because of the synergic effects of probiotics and prebiotics (Al‐Sheraji et al., 2013).

Psyllium seed (*Plantago ovata* Forsk) has been widely distributed in temperate regions of the world, especially in Iran and India (Guo et al., 2009). A highly branched arabinoxylan forming gel mucilage constitutes it that its structure consists of xylose unit arabinose and xylose in the side chains (Fischer et al., 2004). Food and pharmaceutical industries use *Plantago ovata* Forsk seed because of its polysaccharide content (Kaialy et al., 2014). The stability and firmness of natural systems in many products are improved by this polysaccharide and its ability to form a strong gel (Gharibzahedi et al., 2013). Furthermore, *plantago ovata* Forsk as a soluble fiber is used as prebiotic due to its ability to stimulate the growth of bacteria in the digestive system (Rishniw & Wynn, 2011). Physicochemical and sensorial characteristic are two important factors in probiotic yoghurt quality in addition to the survivability of probiotic bacteria; so it is necessary to find a proper compound and its optimum concentration for producing a yoghurt with excellent quality. *P.ovata* Forsk seed mucilage as a new and also natural compound with no unpleasant effects on yoghurt characteristics had a good effect on probiotic microorganism viability and can be used in dairy industries especially in yoghurt production. Based on our knowledge, not many studies have investigated the effect of PFM on the viability of probiotic bacteria, physicochemical, and sensorial properties of yoghurt. Therefore, in this study, the effect of *Plantago ovata* Forsk seed mucilage (PFM) as a prebiotic agent on the survivability of *Lactobacillus acidophilus* and also physicochemical and sensory properties of low‐fat yoghurt samples were investigated.

## 2 MATERIAL AND METHODS

### 2.1 Material

Low‐fat milk (1% fat) and the starter culture containing *streptococcous thermophiles* and *L.delbrueckii* subsp. *Bulgaricus* (CHR HENSEN, Denmark) was prepared from Pegah company (Hamedan, Iran). The lyophilized culture of *L.acidophilus* ATCC 4,356 was obtained from Food microbiology laboratory of the university of Medical Sciences (Hamedan, Iran). The *Plantago ovata* Forsk seed was purchased from the local market in Hamedan province of Iran. All other chemical materials were purchased from Merck Company.

### 2.2 Analysis of *Plantago ovata* Forsk seed

Chemical composition (Lipid, moisture, protein, and ash content) of *Plantago ovata* Forsk seeds was determined by the Soxhlet, hot oven, Kjeldahl and dry‐ash procedures obtained from AOAC (2005).

### 2.3 Extraction of *Plantago ovata* Forsk seed mucilage (PFM)

At first, the impurities of the seed were separated and then mixed in distilled water (water to seed ratio 20:1). The slurry was stirred continuously (1 hr) during the extraction period with magnetic stirrer, then put in laboratory shaker (20 s) to remove the mucilage from seed thoroughly. It centrifuged to remove the remaining seeds (4,000 rpm, 10 min), and pure mucilage was stored in the refrigerator (4 ^0^C) and used for different treatments (Sciarini et al., 2009).

### 2.4 Preparation of *Lactobacillus acidophilus* culture

The culture preparation was done in sterile condition. The cultures in every tube were transferred to tubes containing 10 ml MRS broth and incubated at 37 ^0^C for 18 hr. The tubes containing bacterial suspensions were centrifuged (4,000 rpm, 3 min) to separate MRS broth and settling of the bacterial biomass. After that supernatant was removed, bacterial suspension with 2 McFarland turbidity was prepared by using sterile peptone water (the bacterial count was 6 × 10^8^ CFU/ml) (Krasaekoopt et al., 2004).

### 2.5 Production of yoghurt

Yoghurt samples, including plain yoghurt and probiotic yoghurt as control sample and also probiotic yoghurt containing 0.5, 1, and 2% PFM (T1, T2, T3) were produced in Pegah Company (Hamedan, Iran). The yoghurt samples were made using pasteurized milk (1% fat) of Pegah Company. Different concentrations of PFM (0.5, 1, and 2%) were added to pasteurized milk and after homogenization were put in viscobator. No mucilage was added to the control sample. After cooling to 43 ^0^C, 2% w/w of the starter culture (containing *Streptococcous thermophiles* and *L.delbrueckii* subsp. *Bulgaricus*) and 1% w/w of the *Lactobacillus acidophilus* suspension (2 McFarland turbidity) were added. Samples were packed in containers and incubated at 45 ^0^C for 3–4 hr to reach pH 4.6 and then stored in refrigerator (4 ^0^C). Then, the survivability of *L. acidophilus*, physicochemical, and sensory properties of produced yoghurt samples were evaluated during refrigeration period (0, 7, 14, and 21 days). Each experiment was performed in 3 replications.

### 2.6 Survivability of *L. acidophilus* in yoghurt sample

For enumeration of *L. acidophilus*, at first tenfold serial dilution of each sample was prepared, and then, 0.1 ml of each dilution was cultured on the MRS agar (containing 0.15% bile salt) by surface plating method, and inoculated plates were put in anaerobic jar and incubated at 37 ^0^C for 72 hr. Plates contain 30–300 colonies were counted and reported as CFU/gr (Rezaei et al., 2012).

### 2.7 Quantification of Mold and Yeast

The sabouraud dextrose chloramphenicol agar (SDCA) culture medium was used to investigate mold and yeast in the samples. For this purpose, 0.1 ml of each prepared tenfold serial dilution was plated on SDCA by surface plating method. Inoculated plates were incubated at 25 ^0^C for 3–5 days. Plates contain 15–150 colonies were counted (Fisher & Cook, 1998).

### 2.8 Measurement of pH and acidity

The pH of yoghurt samples was measured during the 21 days of storage period by using a pH meter (Denver, Germany). For measuring titratable acidity of yoghurt samples, 10 g of yoghurt was blended with 20 ml sterile distilled water, and then, it was titrated by 0.1 N NaOH. Phenolphthalein was the indicator, and the acidity was reported based on percentage of lactic acid (Akgun et al., 2016).

### 2.9 Syneresis evaluation

To measure the syneresis of yoghurt samples, 5 g of each sample was put on a separate Whatman paper (NO. 43) setting on a top of a glass container and stored in a refrigerator at 4 ^0^C (2 hr). The weight of the liquid collected at the bottom of the container was measured and reported according to percentage (García‐Pérez et al., 2005).

### 2.10 Water Holding Capacity (WHC) measurement

To obtain the WHC of samples, 5 g of each yoghurt sample was placed in a test tube and centrifuged (4,000 rpm, 30 min). The supernatant was discarded, and the weight of residual precipitate was used for WHC calculating by equation (1):

(1) $$\text{WHC}=\frac{W1‐W2}{W1}\times 100.$$

where w_1_ is the original weight of yoghurt samples, and w_2_ is the supernatant weight (Ladjevardi et al., 2016).

### 2.11 Color evaluation

For measuring yoghurt samples color parameters on different days of storage period, each sample was photographed in a case with white color background. Then, L, a, and b values were determined by adobe photoshop software (7.0.1). The L value represents the brightness, the a value is a position between green and red color, and b value is between blue and yellow color (Hashemi Shahraki et al., 2014; Yam & Papadakis, 2004).

### 2.12 Sensory evaluation

Different organoleptic properties of the yoghurt samples, including flavor (taste and odor), appearance (color and syneresis), and texture, were investigated during the 21 days of storage period by 5‐point hedonic scale method. Treatments were evaluated by 10 panelists (5 men and 5 women, 18–35 years old) and rating ranges from very good to very bad, with scores of 5 (very good) to 1 (very bad) (ISIRI, 1999).

### 2.13 Statistical analysis

Statistical analysis was performed using SAS Software (version 9.1.3) base on a completely randomized design (CRD). The difference between the mean values was determined by one‐way ANOVA and Duncan multiple range test. Significant difference was based on a *p* < .05, and charts were drawn with Excel software (2007). Also, all experiments were done in triplicate.

## 3 RESULTS AND DISCUSSION

### 3.1 Chemical composition of *Plantago Ovata* Forsk seed

Measurement of the general composition of *Plantago Ovata* Forsk seed indicated that the amount of ash, lipid, protein, and moisture content were 2 ± 0.03, 7 ± 0.06, 0 and 6 ± 0.05%, respectively.

### 3.2 Survival of *L. acidophilus*

The obtained results from the effect of PFM on the growth and viability of *L. acidophilus* in produced yoghurt samples during refrigeration period (4 ^0^C) are shown in Table 1. Bacterial count results showed that all samples containing different concentrations of PFM had a higher number of viable bacteria than the control sample and also lower decrease was observed in them during storage period (*p* < .05). Also, the higher concentrations of PFM increased the bacterial count and the highest viability of *L. acidophilus* was observed in produced yoghurt samples with 2% PFM. On the first day of storage period and after incubation, yoghurt samples containing PFM had a higher bacterial count compared to the control sample. The highest (6.68 log CFU/g) and the lowest (6.31 log CFU/g) number of bacterial counts were related to the yoghurt sample containing 2% PFM and control sample, respectively. During the storage period, the decrease in bacterial count in the yoghurt samples containing PFM was significantly lower than the control samples (*p* < .05). The decrease in the number of *L. acidophilus* was 0.2 log in sample made with 2% PFM in the last day of storage period, compared to the first day, while it decreased up to 0.41 log in control sample in 21th of storage period. PFM has some chemical substances such as D‐xylosan, arabinose, D‐galactose, D‐galacturonic, and fibers that considers have prebiotic characteristics (Singh et al., 2011). In fact, during incubation and storage period, *L. acidophilus* used soluble fibers and other nutrients of PFM in yoghurt and boosted their growth and survival. Different studies had shown that there are a lot of substances (different fibers and gums) that can be used as a prebiotic for increasing probiotic viability; for example, Hasani et al. (2016) and (Hasani et al., 2017) reported similar results with the present study in the use of different concentrations of rice bran as a fiber in enhancing bacterial viability. Firooz et al. (2019) investigated the effect of *Plantago Ovata* and Merv mucilage on the survivability of probiotic bacteria and reported similar results with our study. Capela et al. (2006) also indicated that using Raftilose P95 (1.5% concentration) as a prebiotic agent lead to increase in the survivability and growth of *L.acidophilus*, *L. rhamnosus*, *L. casei*, and *Bifidobacterium* during 28 days of refrigeration period.

**TABLE 1:** The *L.acidophilus* number of different treatments during refrigeration period (Log_10_ CFU/g) (Mean ± *SD*)

Treatments	Refrigeration period (Day)			
	0	7	14	21
T1	6.54 ± 0.12^d^	6.40 ± 0.12^e^	6.28 ± 0.13^ef^	6.20 ± 0.20^g^
T2	6.56 ± 0.09^d^	6.48 ± 0.01^dh^	6.33 ± 0.10^o^	6.31 ± 0.12^ofe^
T3	6.68 ± 0.07^i^	6.62 ± 0.03^ijd^	6.55 ± 0.10^d^	6.48 ± 0.05^dh^
B1	6.31 ± 0.21^b^	6.12 ± 0.04^c^	6.01 ± 0.15^ac^	5.9 ± 0.48^a^

Abbreviations: B1: Probiotic yoghurt without PFM. T1, T2, T3: Yoghurt containing 0.5, 1, and 2% PFM.

### 3.3 Number of mold and yeast of treatments

None of the samples showed mold or yeast during 21 days of storage period, which may be due to the sterile conditions of production and maintenance of yoghurt samples.

### 3.4 pH and acidity

**FIGURE 1:**
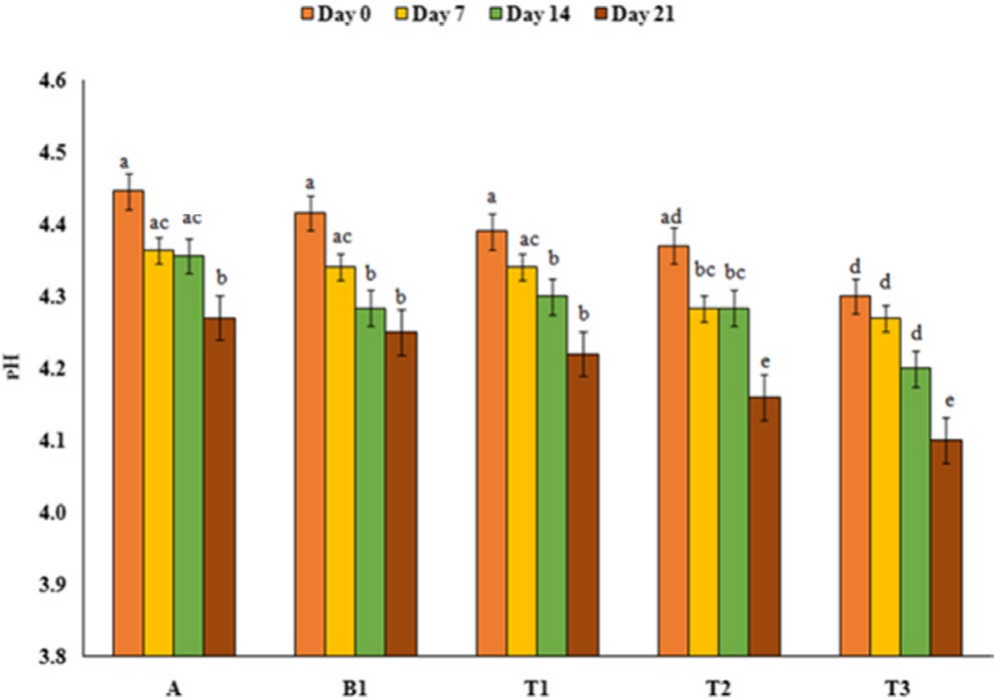
pH evaluation of different treatments during the storage period. A: Natural yoghurt, B1: Probiotic yoghurt without PFM, T1, T2, T3: Yoghurts containing 0.5, 1, and 2% PFM

**FIGURE 2:**
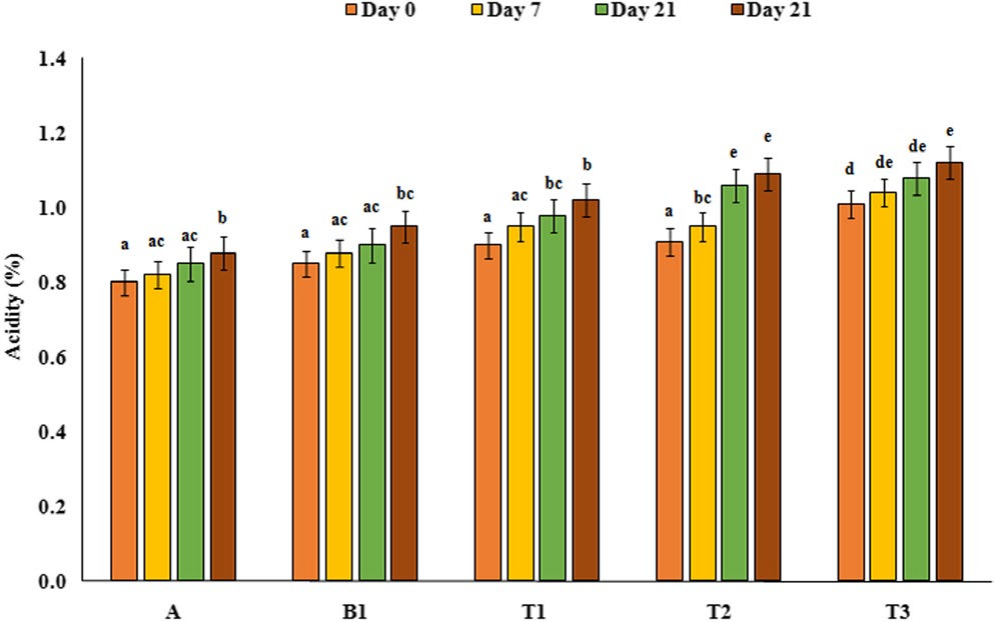
Acidity evaluation of different treatments during the storage period. A: Natural yoghurt, B1: Probiotic yoghurt without PFM, T1, T2, T3: Yoghurts containing 0.5, 1, and 2% PFM

Investigation of the pH and acidity of different samples during storage period showed that there was a relationship between the concentration of PFM and pH and acidity so that treatments contain PFM had lower pH and higher acidity than the control sample, which was significant in the sample containing 2% PFM compared to the others (*p* < .05). Also, over time and on day 21, the pH of all samples decreased and the acidity increased (*p* < .05). It showed that *L.acidophilus* used the substance in the PFM and produced more lactic acid in the samples containing PFM (Figures 1 and 2). This result were similar to the results reported by Kokabian et al. (2020), Hasani et al. (2016), Mousavi et al. (2019a and 2019b), Reyahi‐Khoram et al. (2018), and Curti et al. (2017) that investigated the effect of different natural compounds (grape seed oil, rice bran, flaxseed, mofarrah, and kinova seed powder) on the pH of yoghurt and found that the yoghurts containing this compounds, had lower pH and higher acidity than control sample because of *L.acidophilus* high rate growth. In contrast to our results, Azari‐ Anpar et al. (2017) showed that the pH value and acidity of yoghurt samples were increased and decreased respectively, with the addition of *Aloe vera* foliar gel to the yoghurt sample formulation during the refrigeration period.

### 3.5 Syneresis evaluation result

**FIGURE 3:**
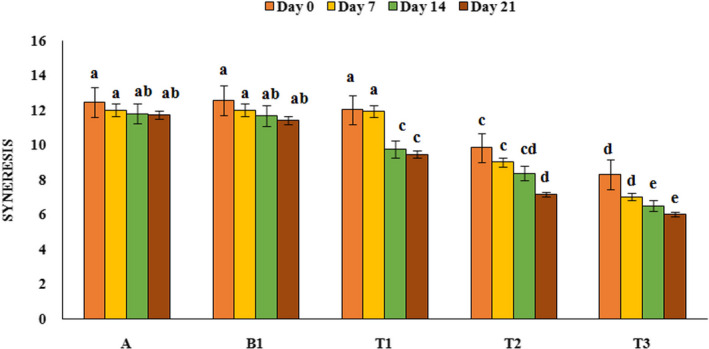
Changes of the syneresis of different yoghurt samples during the storage period. A: Natural yoghurt, B1: Probiotic yoghurt without PFM, T1, T2, T3: Yoghurts containing 0.5, 1, and 2% PFM

Syneresis is an undesirable property of yoghurt and has negative effects on product acceptability. The variation in the amount of different treatments syneresis during storage period is shown in Figure 3. As can be seen, yoghurt samples containing PFM had lower syneresis than control sample and this value was significantly lower in yoghurt samples containing 1 and 2% PFM compared to the other samples (*p* < .05). It seems that the addition of PFM to the yoghurt samples increased osmosis activity and absorbed free water that leads to decrease yoghurt samples syneresis. Also, over the time, the amount of syneresis decreased in treatments containing PFM (*p* < .05) and its reason can be that PFM had more opportunity to absorb much unbound water in the yoghurt. The highest amount of syneresis (12.56%) was observed in probiotic yoghurt without PFM at first day of storage period and the lowest amount was related to yoghurt containing 2% PFM (6%) on 21th day of refrigeration period. Our results were similar to the results reported by other researchers that found adding flaxseed and gundelia puree decreased syneresis in yoghurt (Ebrahimi et al., 2015; Mousavi, et al., 2019a and 2019b). In contrast to the result of this study, Azari‐ Anpar et al. (2017) showed that syneresis was increased gradually with increase in *Aloe vera* foliar gel concentration during the storage period. They stated that the presence of *Aloe vera* gel decrease colloidal stability of casein micelles and so increase yoghurt syneresis.

### 3.6 Water holding capacity (WHC)

**FIGURE 4:**
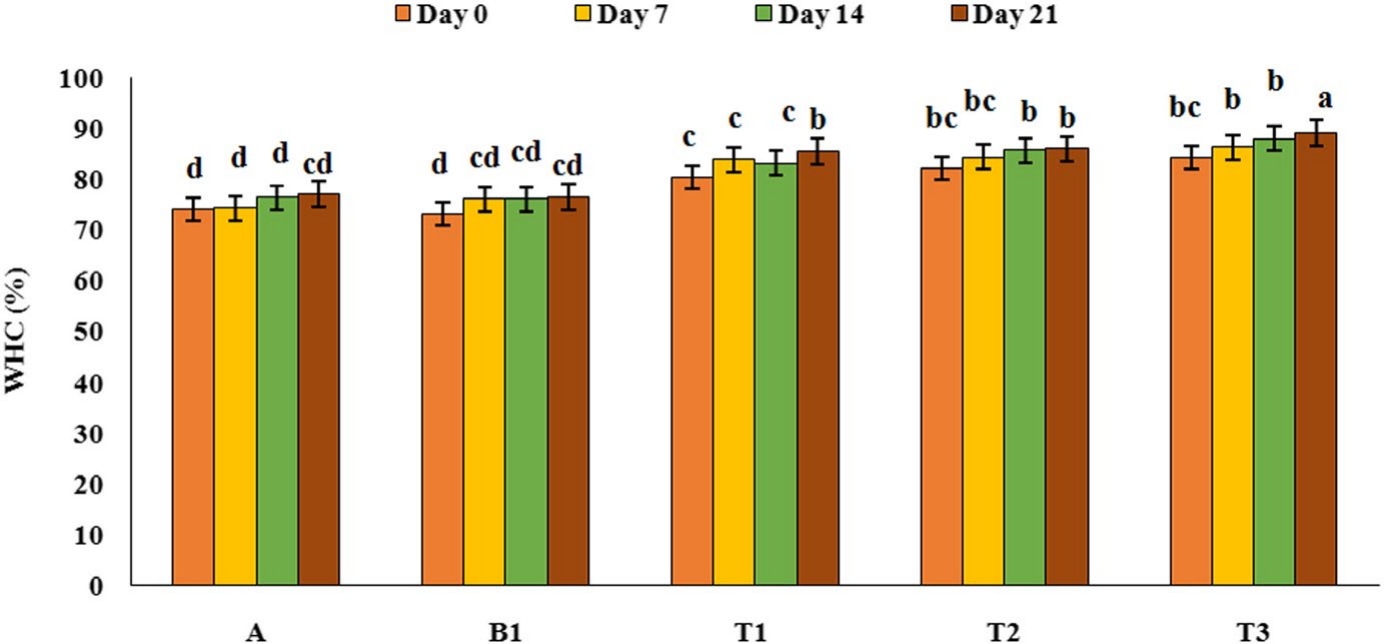
Changes of different yoghurt samples WHC during the storage period. A: Natural yoghurt, B1: Probiotic yoghurt without PFM, T1, T2, T3: Yoghurts containing 0.5, 1, and 2% PFM

WHC is an agreeable characteristic of yoghurt that shows coagulation stability. The results of the WHC changes in different yoghurt samples, during storage period are presented in Figure 4. As can be seen from Figure 4, the samples containing PFM had significantly higher WHC than the control sample and WHC increased with increasing PFM concentration and storage period (*p* < .05). Also, during the storage period WHC increased and samples containing PFM showed the highest WHC on 21th day of refrigeration period (*p* < .05). The highest amount of WHC (89%) was observed in the samples containing 2% PFM on 21th day of refrigeration period and the lowest amount of WHC (73.06%) was related to the probiotic yoghurt sample (without PFM) at the first day of refrigeration period. The higher amount of WHC and stability in the yoghurt samples containing PFM is due to the hydrocolloid characteristic of PFM and its ability to absorb of unbounded water in yoghurt sample. Mousavi, et al. (2019a and 2019b) and Delikanli et al. (2017) reported that by adding flaxseed and milk protein to yoghurt sample, WHC increased significantly (*p* < .05) (Delikanli & Ozcan, 2017; Mousavi, et al., 2019a and 2019b).

### 3.7 Sensory evaluation

**FIGURE 5:**
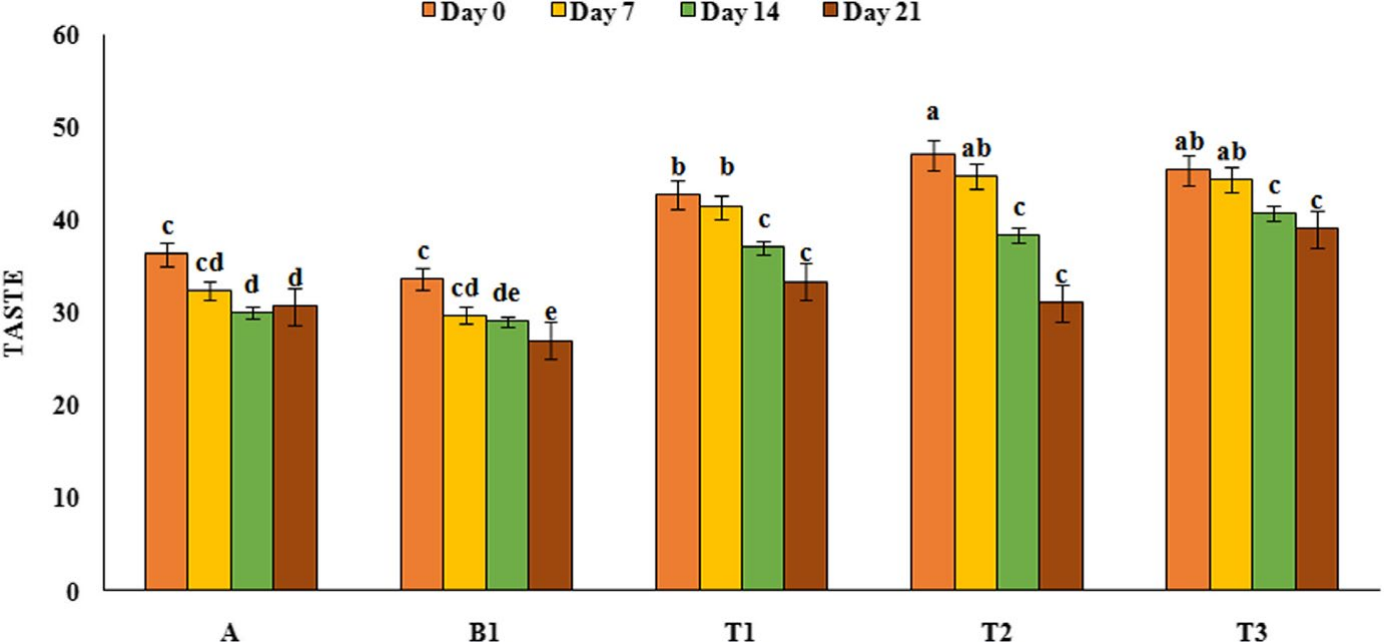
Taste score variation of yoghurt samples during the storage period. A: Natural yoghurt, B1: Probiotic yoghurt without PFM, T1, T2, T3: Yoghurts containing 0.5, 1, and 2% PFM

**FIGURE 6:**
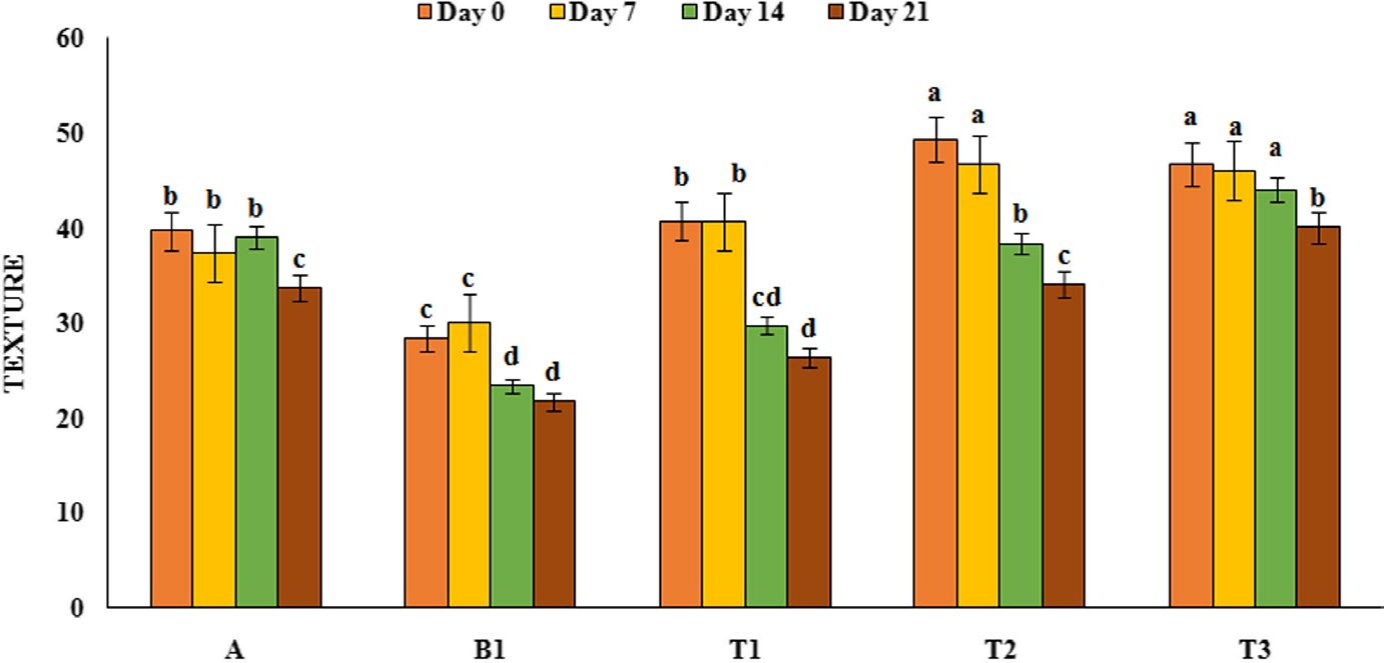
Texture score variation of yoghurt samples during the storage period. A: Natural yoghurt, B1: Probiotic yoghurt without PFM, T1, T2, T3: Yoghurts containing 0.5, 1 and 2% PFM

**FIGURE 7:**
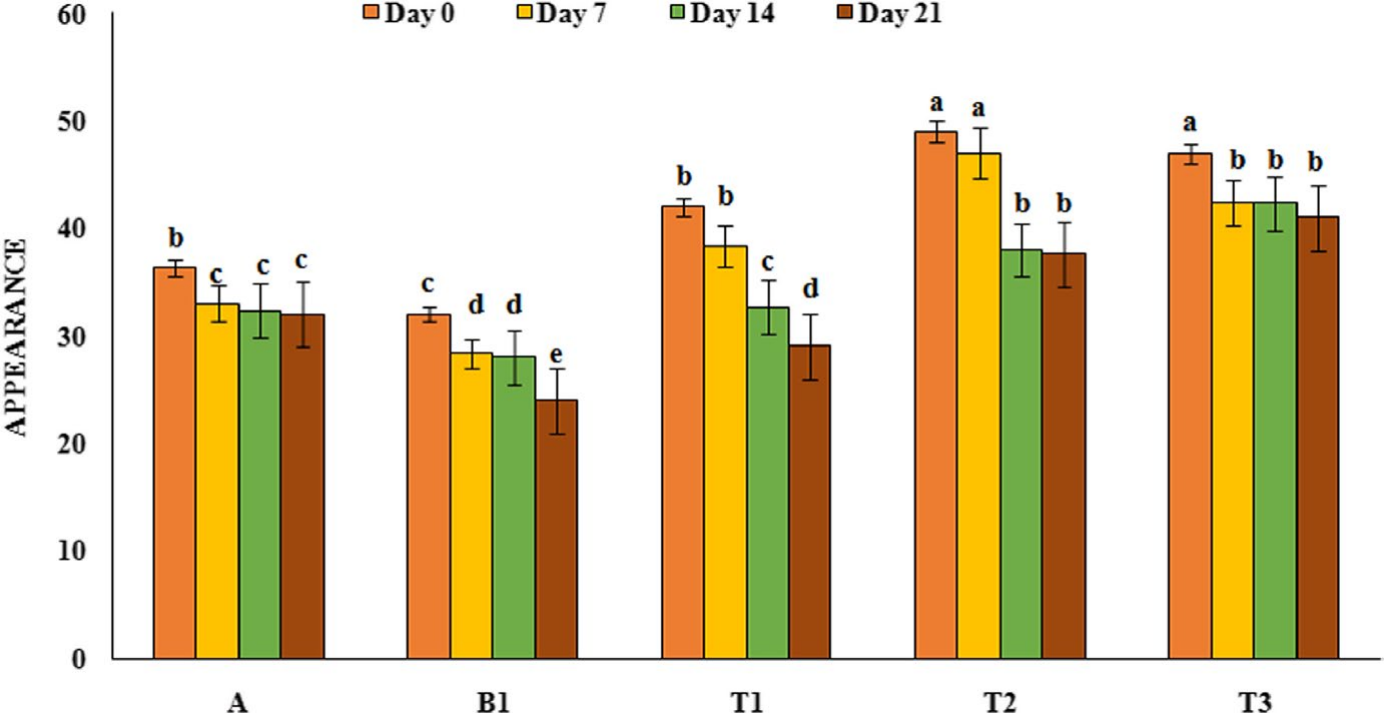
Appearance score variation of yoghurt samples during the storage period A: Natural yoghurt, B1: Probiotic yoghurt without PFM, T1, T2, T3: Yoghurts containing 0.5, 1, and 2%

Sensory properties are an important feature for consumers and society acceptance, and a product with no desirable sensory properties will not be consumed in public even if it has high nutritional value. In this study, yoghurt samples were evaluated for taste, texture, and appearance and their results are shown in Figures 5, 6, and 7 respectively. Sensory analysis of the taste by panelists indicated that treatments containing PFM had higher taste score than control sample and the highest taste score was observed in yoghurt samples containing 1% PFM (47) at the first day of refrigeration period. Also, during the refrigeration period, taste scores in all yoghurt samples decreased (*p* < .05), which could be due to the decrease in pH and increase in lactic acid content. Results of texture analysis showed that yoghurt sample containing PFM had better texture and higher score compared to control sample and over the time the scores of texture of all yoghurt samples decreased (*p* < .05). The highest texture score (49.33) was related to yoghurt sample containing 2% PFM while the lowest texture score (28.33) was related to the probiotic yoghurt sample without PFM. Evaluation of samples in terms of appearance showed that yoghurt sample containing PFM had higher appearance scores than the control sample (*p* < .05) and over the time, on day 21, the appearance scores of all yoghurt samples decreased. Probiotic yoghurt contains 1% PFM had the highest appearance score (49) compared to the others. Our results was similar to the results reported by Ebrahimi et al. (2015), Domagala et al. (2006), and Sanz et al. (2008) that showed by addition of gundelia, oat‐ maltodextrin and asparagus fiber to the yoghurt samples, sensory scores increased and this substances had positive effect on texture, appearance, and taste of produced yoghurt samples.

### 3.8 Colorimetry

**FIGURE 8:**
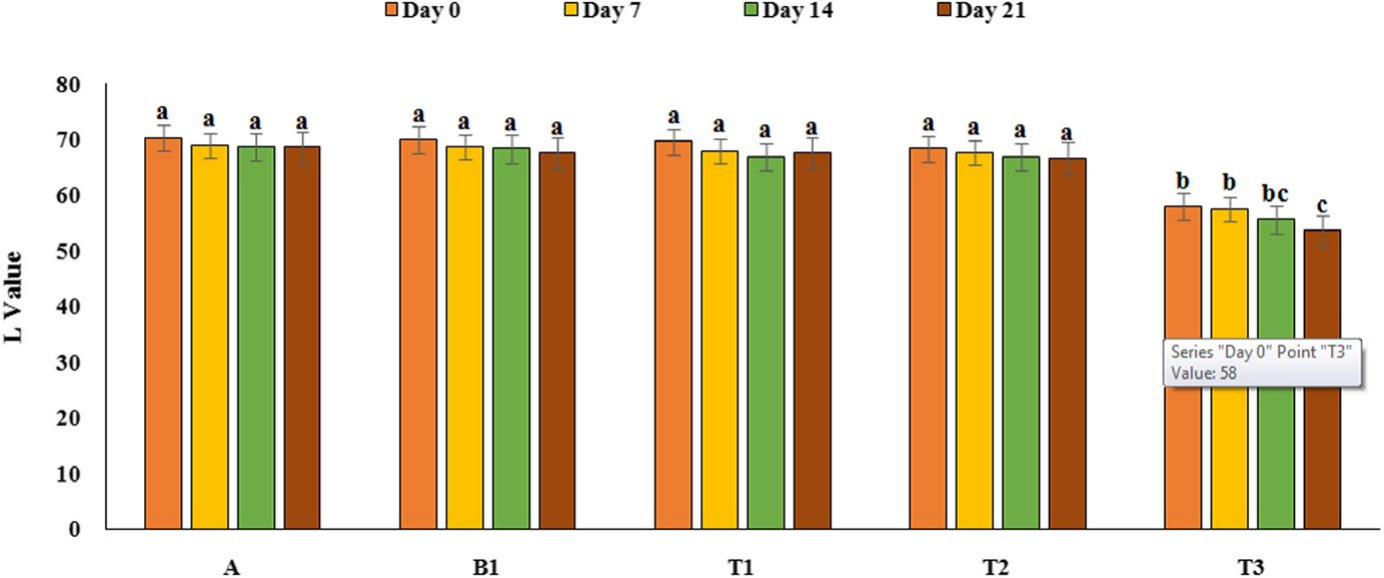
Variation of L value of yoghurt samples during the storage period. A: Natural yoghurt, B1: Probiotic yoghurt without PFM, T1, T2, T3: Yoghurts containing 0.5, 1, and 2% PFM

**FIGURE 9:**
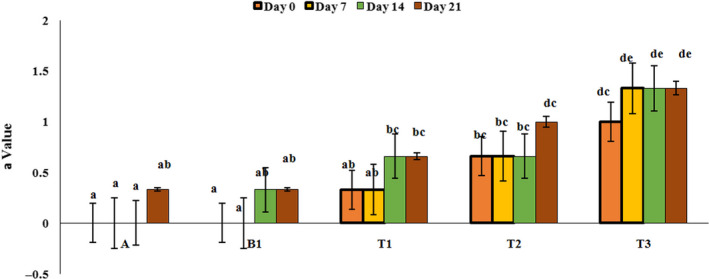
Variation of a value of yoghurt samples during the storage period. A: Natural yoghurt, B1: Probiotic yoghurt without PFM, T1, T2, T3: Yoghurts containing 0.5, 1, and 2% PFM

**FIGURE 10:**
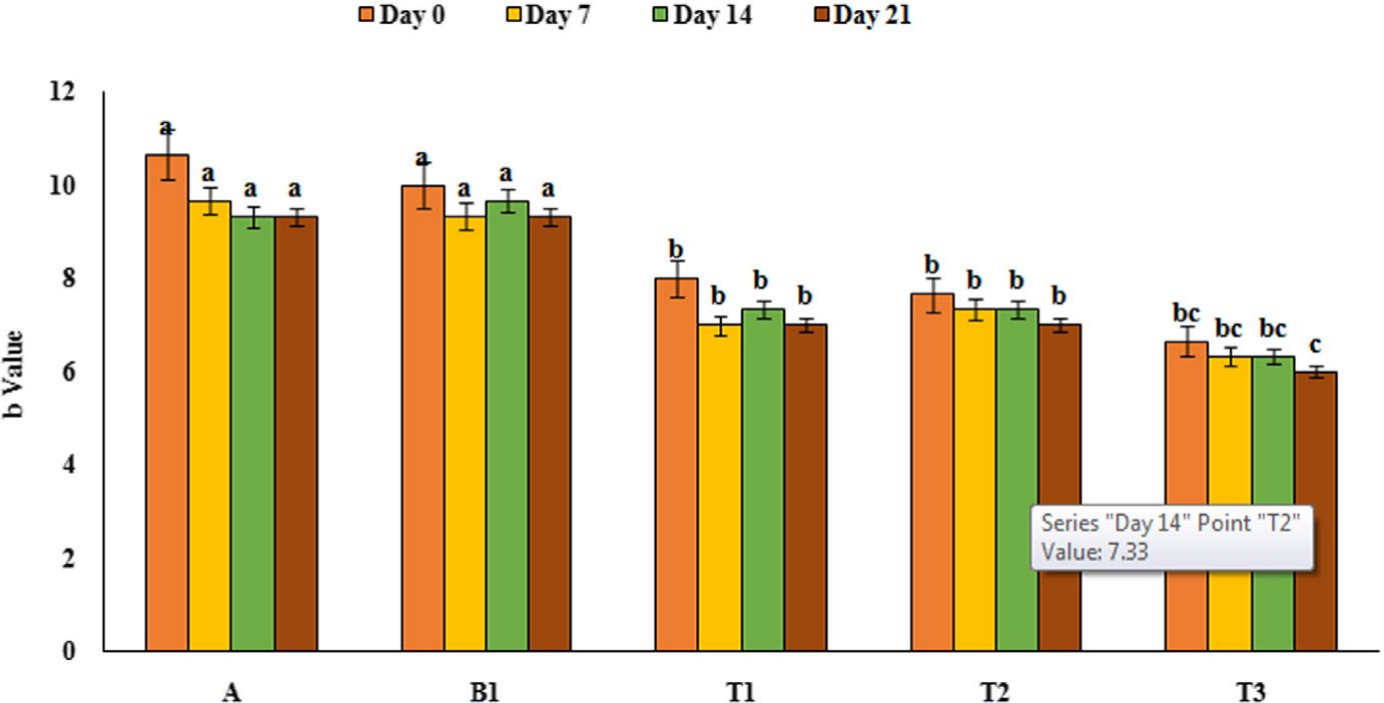
Variation of b value of yoghurt samples during the storage period. A: Natural yoghurt, B1: Probiotic yoghurt without PFM, T1, T2, T3: Yoghurts containing 0.5, 1, and 2% PFM

In order to evaluate the color parameters of yoghurt samples, L, a, and b values were investigated by photoshop software (Adobe photoshop CC, 2018) and the results are presented in Figures 8, 9, and 10. As can be seen from Figure 8, only yoghurt sample contains 2% PFM had lower L value than the control sample (p‌<0.05), and over the time, on 21th day of refrigeration period, L value decreased significantly (in yoghurt containing 2% PFM). It could be due to the color of mucilage that in high concentration affect the L value of yoghurt samples. Results of a factor (green and red color) investigation showed that yoghurt samples containing PFM especially yoghurt containing 2% PFM had higher a value compared to control sample (reddish) (*p*<‌0.05). Also, results of b value showed that yoghurt samples containing PFM had lower b value than the control sample (p‌<‌0.05). Also, time had no significant effect on the a and b value in all treatments. Staffolo et al. (2004) reported similar results and indicated that yoghurt samples containing different fibers (apple, wheat, and inulin) had different color parameters compared to the control samples and by adding these substances, L value decreased (Staffolo et al., 2004). Also, García‐Pérez et al. (2005) reported that during the storage period, brightness of yoghurt samples decreased and red color increased that can be due to decrease in pH (García‐Pérez et al., 2005). Mousavi, et al. (2019) observed an increase in a value in flaxseed‐enriched yoghurt compared to the control sample that can be due to the pigmentation of the flaxseed (Mousavi, et al., 2019).

## 4 CONCLUSION

The results of this study showed that PFM had a positive effect on growth and survivability of *L.acidophilus*, and there was a direct correlation between PFM concentration and *L.acidophilus* in probiotic yoghurt. According to this finding, using PFM in yoghurt samples improved different physicochemical properties such as WHC, syneresis, texture, and addition 2% PFM to yoghurt samples impart the best characteristic in the final yoghurt product. Also, PFM had no adverse effect on sensory properties of yoghurt sample and even yoghurt containing 1% PFM had the best taste and appearance. In general, the use of PFM can be a suitable for producing probiotic yoghurt and 2% concentration of PFM lead to the best results for *L.acidophilus* viability and physicochemical properties while 1% PFM is the best choice to reach the best color and appearance beside other characteristic.

## 5 ETHICAL REVIEW

This study does not involve any human or animal testing.

## CONFLICT OF INTEREST

The authors declare that they do not have any conflict of interest.

## INFORMED CONSENT

Written informed consent was obtained from all study participants.
